# Preservice teachers’ views of the nature of science using Black heritage, lived experiences, and science capital as a context for inquiry

**DOI:** 10.3389/fsoc.2026.1814225

**Published:** 2026-08-06

**Authors:** Catherine L. Quinlan

**Affiliations:** Department of Curriculum and Instruction, School of Education, North Carolina Central University, Durham, NC, United States

**Keywords:** Black preservice teachers, cultural relevance, culturally sustaining inquiries, equity and inclusion, family resemblance approach, nature of science (NOS), schema theory, science curriculum

## Abstract

This research is part of a larger project to situate Black lived experiences and narratives in the science curricula. This work provides further analyses and conceptualization of a portion of prior work. In this study, Black science capital serves as a context for inquiry explorations into scientific, science-related, and non-science inquiries, depending on preservice teachers’ interests. Participants were undergraduate elementary education majors enrolled in a second-semester science methods course at an Historically Black College and University (HBCU). Pre- and post-surveys consisted of two types of questions. Selected questions from the widely used, already-vetted Lederman et al. views of the nature of science, version C, were combined with new questions about including Black representation in the science curricula. An amalgamation of frameworks was used to facilitate connections with science, the nature of science and knowledge, and preservice teachers’ cultural and cognitive predispositions for science, within a context that focuses on Black assets. The family resemblance approach provided surface connections between the nature of science and outcomes related to the lived experiences and science capital of Black heritage in science, while schema theory attended to nuances and connections that emerged from different types of knowledge. The significance of this work lies in the inclusion and use of data for inquiry, which captures the science capital of Black African heritage. Recommendations for future directions and improvements are provided for continued discussions, with implications for connections between scientific ways of knowing, practices, and approaches that meaningfully include the practices and experiences of Black with Indigenous people, to provide a more accurate and comprehensive view of science.

## Introduction

The need to attend to the content and context that influence perspectives, ways of being, and the epistemology of knowing continues to be important. Research in science education shows that the history of science provides a context for understanding aspects of the nature of science (Fouad et al., 2015; Nouri and McComas, 2021). However, this courtesy does not extend to the narratives, lived experiences, and heritage of Black or Indigenous people, which are not viewed as sources of understanding about the nature of science. The continued impacts of this type of exclusion from the knowledge base, which defines the epistemology of science and what is worthy of knowing, contribute to the continuing negative social schemas about Black people. The American Psychological Association defines social schema relative to individual schema as:

a cognitive structure of organized information, or representations, about social norms and collective patterns of behavior within society. Whereas a self-schema involves a person’s conception of herself or himself as an individual and in terms of a particular personal role (or roles) in life, social schemata often underlie behavior of the person acting within group contexts—particularly larger group or societal contexts (American Psychological Association, 2024).

What this means for Black students is that the science context does not take advantage of their individual schemata, and so they are less likely to make connections with their Black heritage, plight in life, or lived experiences. This contrasts with White students, who have the opportunity to connect with White representations and culture within Western science, whether knowingly or unknowingly. The omission of Black and other Indigenous ways of knowing is a missed opportunity to engage in critical conversations with knowledge that is located outside the purview of Western knowledge or Western ways of knowing.

This research used content that situates Black heritage and lived experiences in the science curricula. The science content provided a context for learning about the nature of science using inquiry explorations and Black science capital as data and context. This study was guided by the research questions: How can Black science capital, assets, heritage, and lived experiences provide a context for understanding the nature of science? How do preservice teachers’ (PST) views of the nature of science change because of their engagement in inquiry using Black science capital? What are the advantages and disadvantages? What do PSTs learn about including Black heritage, science capital, and lived experiences in the science classroom?

The importance of this research is underscored by works which showed that narratives and histories provided a context for understanding aspects of the nature of science (Fouad et al., 2015; Nouri and McComas, 2021). However, since Black lived experiences and heritage knowledge are rarely, if ever, included in the curricula (King and Swartz, 2018), much less associated with science, the content for this research draws from Black science capital and its resemblances with science. Thus, another significance of this work is an opportunity for “‘re-membering’ or reconnecting knowledge of the past that has been silenced or distorted” (King and Swartz, 2016, p. 1). Re-membering returns and provides contexts and histories that are neither used to ground science nor provide other perspectives. In this study, Erduran and Dagher (2014) family resemblance approach (FRA) is used as a framework for situating this context in science, and Marshall’s (1995) schema theory is used as a framework for making connections with the lived experiences of Black heritage.

## Literature review

This literature review uses a theoretical approach to examine the nature of science and other research literature. This approach focuses on vetting the use of Black heritage as a context for exploring the epistemology of science learning. This literature review is framed using ideas from motivated thinking—the idea that the heart can guide the mind (Molden and Higgins, 2005)—and the family resemblance approach (FRA) to provide a rationale for previous and proposed directions in understanding the nature of science.

Irzik and Nola (2014) introduced the term “‘family resemblance approach’” (p. 1000) to describe categories that provide a consensus on the nature of science. The goal of their ‘family resemblance approach’ was to create a ‘systematic and unifying perspective’ (p. 1003) that offered a more integrative and comprehensive take on the nature of science by combining themes and perspectives from researchers in science education. They suggested “begin[ning] with a broad distinction between *science as a cognitive-epistemic system of thought and practice* on the one hand and *science as a social-institutional system* on the other hand” (p. 1003). Irzik and Nola’s philosophy was that the categories, themes, or perspectives should not function as discrete entities divorced from their context. They tabulated eight categories of science, which included “1. Processes of inquiry; 2. Aims and values; 3. Methods and methodological rules; 4. Scientific knowledge,” (Irzik and Nola, 2014, p. 1009) as part of the cognitive–epistemic system, and“5. Professional activities; 6. Scientific ethos; 7. Social certification and dissemination of scientific knowledge; 8. Social values” (Irzik and Nola, 2014, p. 1009) as part of the social system. While they acknowledged that this was not comprehensive, they highlighted the need to explore exactly what was meant by “nature” or “nature of science.”

**Figure 1:**
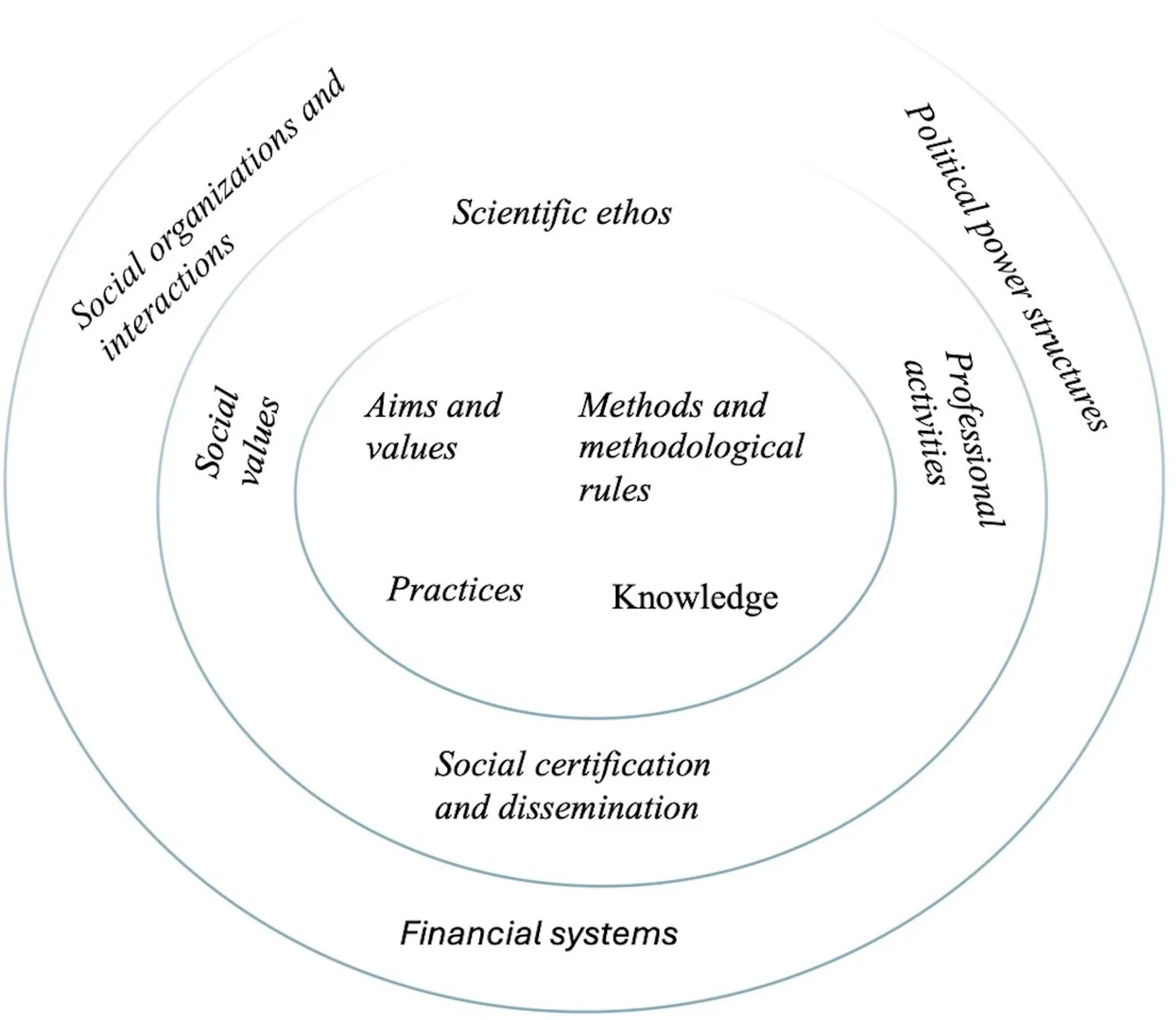
Adoption of Erduran and Dagher’s (2014, p. 28) conceptual model of the family resemblance approach (FRA) with permission from author.

Erduran and Dagher (2014) expanded on Irzik and Nola (2014) ‘family resemblance approach’ by adding to the categories and creating a conceptual model that they believed had more practical applications and offered flexibility to the field of science education (see Figure 1). This conceptualization, known as the family resemblance approach (FRA) in science education, consisted of three concentric circles with “aims and values; methods and methodological rules; practices; knowledge” at the center. Next were “scientific ethos; professional activities; social certification and dissemination; social values” (p. 28). The outermost circle contained “social organizations and interactions; political power structures; financial systems” (Erduran and Dagher, 2014).

Erduran and Dagher (2014) noted that their expanded categories would ensure that all sciences were included, allowing teachers to determine how the nature of science (NOS) was relevant to what they were already doing, rather than needing to change their practices to be confined to a discrete set of categories.

The FRA supports the need for more comprehensive approaches that consider the social context alongside cognition when looking at the NOS. Erduran (2014) noted that a more holistic approach would help students understand the development of scientific knowledge and practices, which would assist in their understanding of the atom. Researchers agree that science and “nature of science” research focus more on the cognitive–epistemic system dimension than on the social-institutional system dimension, as if science occurred in a vacuum or had no social context or history (Erduran, 2014; Irzik and Nola, 2011). In Erduran and Dagher’s (2014) FRA, the cognitive–epistemic dimensions are defined by four main categories: aims and values, scientific practices, methods and methodological rules, and scientific knowledge (Erduran, 2014; Erduran et al., 2019; McDonald, 2017; Takriti et al., 2022). Erduran et al. (2019) defined the social-institutional system dimension using seven categories expanded from Irzik and Nola (2011) four categories, namely, professional activities, scientific ethos, social certification, social values of science, social organizations and interactions, political power structures, and financial systems (p. 316), which make up the social activities of science. Research shows increasing use of FRA to understand NOS studies in science education (Cheung and Erduran, 2022) as a more holistic approach to NOS (Dagher and Erduran, 2016, 2017). Wu and Erduran (2022) found alignment between the FRA and scientists’ perspectives, and Erduran (2022) noted that FRA and NOS could be reframed for beneficial philosophical arguments using empirical and methodological data, and for inspiring creativity. Irzik and Nola (2023) later modified and expanded the categories of the cognitive -epistemic system to include “Practices of inquiry”; “aims and epistemic values”; and “scientific knowledge or belief” (p. 1234) and their social-institutional categories to include “Non-epistemic social values”; “Reward structure”; “Social Organization”; “Power structure”; and “Economics of science” (p. 1234).

Including the lived experiences and narratives of Black heritage could provide a more holistic approach that considers the cognitive and social dimensions of science. The experiences of Black scientists and Black people in STEM generally underscore the importance of *professional activities* and *social certification* as *dimensions* of the FRA that serve as gatekeepers of science and lead to a lack of recognition of Black scientists (Brown et al., 2016; Carlone and Johnson, 2007). The norms of the *scientific ethos* do not apply fairly to the experiences of Black people, prompting researchers such as Felicia Moore Mensah (Moore, 2007) to advocate for educating minorities about these gatekeeping mechanisms. Erduran (2014) noted that “by scientific practices, [she] refer[ed] to those practices that lead to the generation, evaluation and revision of scientific knowledge” (p. 39). This knowledge could extend to the contributions, lived experiences, and heritage of Black people, whose contributions to Western science or food systems often go unacknowledged by science and society (Quinlan, 2024a, 2024b; Carney, 2001).

Put another way, Kuhn (1996) described this social dimension of science as the scientific community of practice, which debates and serves as the gatekeepers of scientific knowledge. Irzik and Nola (2011) described the perpetuation of confined ideas about what science is as the “consensus” view, which “portrays a too monolithic picture of science and is blind to the differences among scientific disciplines” (p. 593), pointing to examples of non-experimental science disciplines. Researchers highlighted the importance of context when looking at the nature of science by pointing to the role of theoretical scientists, which did not involve lab experiments (Wong and Hodson, 2009), and to the context-specific responses of the scientists in Sandoval and Redman’s (2015) study. Irzik and Nola advocated for the use of the FRA, while cautioning that just because a discipline displayed some aspects of the FRA, this did not mean that it was science, as other non-science disciplines make observations and inferences, for example. Including the cultural and social assets of Black heritage and lived experiences can provide new context where hidden figures were previously left out of texts (Quinlan, 2024a, 2024b).

### Social and cognitive dimensions of NOS

Generally, NOS research focuses on the cognitive–epistemic dimension by looking at changes in students’ views of the tenets of the nature of science. While different approaches are used (Burgin and Sadler, 2016), the nature of information does not provide an opportunity to understand the nature of scientific experiences for Black people in the United States or around the world, nor scientific knowledge that is supported by the cultural knowledge of Indigenous or Black people in the United States.

Hanuscin et al. (2006) looked at the NOS views of undergraduate teaching assistants (TA) in physical science laboratories for PSTs, in which their role as TAs served as an explicit and reflective approach to NOS. Schwartz et al. (2004) used inquiry as an approach to developing “appropriate” views of the nature of science. They described inquiry itself as a “context” for learning about the nature of science, and a social and cultural view of the nature of science (VNOS) was exemplified by the following task:

Examine and describe the immediate social and cultural community of your laboratory setting. Do you think everyone makes the same assumptions and has the same general direction for the research (i.e., are the projects related and collaborative?)? If possible, attend some journal club meetings to expand your description of the scientific community. Be observant to occasions where other research communities are discussed. Is the science in your lab considered ‘local’ or ‘global?’ (Schwartz et al., 2004, p. 640).

Similarly, an earlier study by Bell and Lederman (2003) looked at professors’ and research scientists’ views of NOS and their decision-making for different issues using the earlier VNOS-B questionnaire. The results showed that two of the five scenarios generated responses characterized by social and cultural embeddedness. For scenario 1, “Fetal tissue implantation,” respondents displayed “moral/ethical issues,” “values,” and “social/political issues.” Scenario 2, “global warming and greenhouse gas emissions,” responses were characterized as “social/political issues” and “economics (cost/benefit).” The remaining scenarios were given the category “value.” These included scenario 3, “diet, exercise, and cancer,” and scenario 4 “cigarette smoking and cancer.” In making the connection between scientific evidence and their own decision-making, one individual explicitly stated:

So, I think that as a citizen, you take the scientific information, but then you also have to make decisions based on values and societal, cultural, and personal goals. And that’s true of any sort of everyday decision that relates to science and technology. I do not think anybody takes scientific information, whether it’s equivocal or unequivocal, and incorporates it whole clause into their everyday experience. (A5, DMQ-interview) (p. 366).

While NOS tenets encouraged a focus on various aspects of science, the social and cultural NOS was highlighted here to emphasize the influence of social contexts on the *aims and values* or on the cognitive–epistemic aims of science and science education.

Similarly, the sample responses included with the Views of Nature of Science, version C (VNOS-C) instrument (Lederman et al., 2002) displayed very general ideas about culture, especially for those characterized as “naïve” views. However, these connections could also be characterized as Eurocentric, as is to be expected, because they pointed to bigger ideas that have been promoted by Western science or by the consensus view. This is supported by research using FRA which shows textbook examples that reflect *social utility* and that the concept of “race” supports representation and reproduction of White Western scientists and culture (Reinisch and Fricke, 2022). For example, one response characterized as an informed social and cultural view stated that: “On a final note, just like science alone cannot and should not provide society with a definitive answer regarding when human life begins; it cannot and should not alone decide if and how fetal tissue should be donated. (A5, DMQ)” (Bell and Lederman, 2003, p. 359). Other VNOS labeled as informed views included the following examples:

Of course culture influence the ideas in science. It was more than a 100 years after Copernicus that his ideas were considered because religious beliefs of the church sort of favored the geocentric model.

All factors in society and the culture influence the acceptance of scientific ideas. Like the theory of evolution was not accepted in France and totally endorsed in Germany for basically national, social, and also cultural elements (Lederman et al., 2002, 516).

These examples focus on theories or Western knowledge or information that were not accepted. The plight of people who are not fully accepted into scientific environments today and the origins of knowledge still not acknowledged do not reflect the social or cultural views espoused in nature of science research. Though historic, they exemplify what Bourdieu (1977) describes as the symbolic capital, and social and cultural capital promoted and valued by society today.

### History of science as a context

Lastly, Fouad et al. (2015) used history through storytelling, which accompanied activities for elementary students, to look at their NOS views. One example included “the story of Archimedes and the Crown Problem elicited a number of questions about the historical figures, such as how Hiero became king and how Archimedes died” (p. 1123). Another included the biography of Jabir (Ja ¯bir) to discuss the various experiments he carried out. When comparing students’ VNOS from the inquiry group and the history of science group, the students in the history group progressed more in their understandings about the nature of science. This means that the history of science provided access to nature of science understandings compared with just carrying out inquiry alone. History of science storytelling provided access, inviting questions and curiosities from the children. Fouad et al. (2015) noted that students had difficulty understanding the idea of a social and cultural context for science. The teacher asked, “Did everybody have the same idea? No, we did not, did we? We were all looking at the same information, why?” (p. 1129). These questions were asked to help students articulate these ideas.

Similarly, the intervention in this study on Black representation provided an opportunity for several approaches—inquiry, explicit, implicit, reflective. However, engagement depended on students’ choice of topic for exploration and the degree of relatedness to science or other contexts. The use of Black heritage and lived experiences during inquiry was also exploratory. This study explored whether inquiries using Black heritage and lived experiences could be a context not only for understanding the tenets of the nature of science, but as an explicit approach to understanding social and cultural embeddedness without explicit mention of NOS or NOS tenets. This study sought to understand whether preservice teachers’ (PSTs) changes in views could also indicate whether Black narratives could inform the social and cultural embeddedness of science.

### The significance and uniqueness of this work

The significance of this work was not only in considering the importance of context and content but also the source of data and approaches that influence students’ experiences. Implicit was the consideration that data source and context mattered not only in the kinds of questions students might be prompted to explore but also in the lenses they used to capture their understandings. This was based on several assumptions. First, that pre-existing science content did not provide optimal potential to learn, “where a problem makes use of and extends the language, knowledge, motivation and relationships already available to learners” (Enciso and Ryan, 2011, p. 133). These relationships could be thought of in terms of the influence of social, cultural, political, and historical underpinnings. The context would provide a new way of thinking about data, thinking about thinking, and provide an opportunity to tap into Black students’ prior experiences and cultural underpinnings.

Second, the data source used gave students the opportunity to explore questions they would not normally have the opportunity to generate in science. One significance of the data source is seen in the background information provided by the National Research Council (NRC) authors (National Research Council, 2008; National Research Council, 2006; National Research Council, 1996) from which the data was derived. These NRC researchers noted the negative characterization and misclassifications that led to crops being described as “inferior” and “unworthy.” They noted that the instances that led to these “misunderstandings” were also caused by flawed scientific conclusions, such as comparing crop performance on poor versus rich soils. With these predispositions, the scientists and researchers documented and captured the abundant crops of Africa, providing an opportunity to learn about African heritage from scientific, social, cultural, historical, and other perspectives (Quinlan, 2024b). The data provided information about these crops, giving students the opportunity to ask questions, perform analyses, and interpret different kinds of data that would not normally be used in Westernized classroom curricula. The significance of this work is in the essence of inclusion and Indigenous voice, which allows cultures to be essential to their own development, to speak for and define themselves (King and Swartz, 2018).

## Methodology

The methodology provides insights into the research design, participants, data collection, and data analysis. This research was part of a larger pragmatic qualitative inquiry using content that is new to the field of science education (Quinlan, 2021). Therefore, attention was given to the limitations, validity, and reliability in the methods, findings, and discussion sections.

### Research design

#### Participants

Participants were nine female undergraduate preservice elementary education majors enrolled in a compulsory junior science methods course, reflecting a convenience sampling. The course was conducted entirely online during the COVID-19 pandemic. Institutional Review Board approval was obtained. Nine out of 12 preservice teachers (PSTs) signed consent forms agreeing to participate and permit use of course artifacts. Participants were asked to write descriptors to identify themselves ethnically and racially. Three PSTs described themselves ethnically and racially as both African American and Black. Other descriptors included Black; African American; ethnically Ethiopian and racially Black; Caribbean heritage from the island of Trinidad and Tobago and African American; and Black and White. Overall, seven out of nine used Black in their descriptor and five out of nine used African American in their descriptor. This delineation was important in a historically Black college setting, reflecting the diversity among Black/African American populations and peoples of Black heritage. Participant-generated pseudonyms are used in this paper.

#### Overview of implementation

Implementation was embedded in the second of two required science methods courses taken by elementary education majors. The implementation took place mainly in a synchronous live format, which was recorded. Course instruction prepared PSTs for their capstone assignments, one of which consisted of creating lesson plans using the 5E instructional model—Engagement, Exploration, Explanation, Elaboration, and Evaluation (Bybee et al., 2006)—as a lesson format. The course was divided into three main sections: 1. Introduction to inquiry and scientific practices; 2. 5E model inquiry exemplars for Black cultural representation; and 3. Using the 5E model and the Next Generation Science Standards (NGSS) (NGSS Lead States, 2013) to create lesson plans (see timeline in Table 1).

**Table 1:** Overview of timeline for pre- and post-questionnaires and Black representation implementation.

Topic	Objectives	Inquiry/5E/
Week 1 – Pre-VNOS-C questionnaire		
Introduction to Inquiry and the Scientific Practices		
Introduction to spatial thinking. Inquiry in science and in the classroom	Inquiry as a scientific practice Week 2 - Introduction to Black representation in the Science Curriculum	What is inquiry? Why is it important? What does inquiry look like in the classroom?
Week 3–4: 5E Model inquiry exemplars for Black cultural representation		
Cultural representation of African Americans in the STEM Curriculum	Using data that represents Black culture to explore a science topic Introduction to using the lived experiences and narratives of African Americans to explore science	How to use data as evidence during teaching and during data collection for your science content topic. Use link to data resources to explore your science topic on cultural representation
	Hands on Activity: Use of African Rock Art to Learn about Chemistry	Generating ideas for research question, planning, data collection and analysis. Articles by Quinlan
Week 5: Student presentations on science content explorations		
Using the 5E Model and the Next Generation Science Standards to Create Lesson Plans		
Week 13 – Post VNOS-C and Black representation questionnaire		

The course began with a discussion about inquiry and an introduction to Black representation in the science curriculum. Since this also marked the beginning of PSTs’ short-term science inquiry exploration task, they were introduced to ideas about inquiry as a pedagogy in science. The PSTs engaged in discussions about the Gullah/Geechee African American people. (Gullah/Geechee African Americans lived in isolation in what used to be malaria-ridden coastal areas or Gullah/Geechee islands off the southeastern coasts, affording them a unique opportunity to retain African customs.) Their content folders on the Blackboard platform contained video clips and an animation, created as part of this larger project on Black representation, which were used as an engaging context and content. The titles of the clips reflected the topic and content and included *Native Practices and Plants that Heal*, *Oyster Beds and Tabby Sustainability Practices,* and *Food Adaptations and the Making of Sugar*. The making of and considerations for these video clips and animation are described in a recent publication on emergent themes and pragmatic research methodologies (Quinlan, 2021). The PSTs were given a reflection guide with questions that fell under three categories: main ideas, what they found surprising, and what they found confusing.

The inquiry exploration was framed by the 5E model, in which PSTs generated questions from the “engage” activities and from looking at the data cards created for this project. The data cards were created using research from the National Research Council (NRC) scientists’ (National Research Council, 2008; National Research Council, 2006; National Research Council, 1996) work on the *Lost Crops of Africa* (Quinlan, 2024b). The data cards contained a summary of science connections as interpreted by a graduate assistant who was a non-science major and worked briefly with the researcher. Additional links were provided as supplemental data sources, and PSTs were encouraged to pursue additional sources if needed. The content developed for the data cards has implications for the NGSS, as indicated in a recent publication on framing and determining content and standards for including Black lived experiences and narratives (Quinlan, 2024b). The PSTs also engaged in brief, hands-on inquiry explorations on African rock art image analysis (Quinlan, 2019a, 2024a). They participated in a brief exploration in small groups during a class activity in which they learned about African rock art, the people behind the art, and did a hands-on activity using household materials to explore which materials would make the best liquid, color, and binder combinations for paint.

### Data collection

Several data sources were used to provide “rich, thick descriptions to convey the findings” (Creswell and Creswell, 2018, p. 2000). Data collection consisted of student artifacts within the course. A pre- and post-questionnaire was administered to the PSTs. The questions drew from 8 out of 10 of the Views of the Nature of Science Questionnaire version C (VNOS-C) (Lederman et al., 2002) to shed light on PSTs’ understandings related to the nature of science and the epistemology of science (see questionnaire in supplemental). The VNOS-C has been repeatedly validated by science education researchers (Hanuscin et al., 2006; Lederman et al., 2002; Schwartz et al., 2004). The questions specific to scientists’ certainty about the structure and evidence for atoms, and scientists’ certainty about and evidence for what a species is, were deleted from the questionnaire. As a researcher who is also of Black heritage, this research was particularly biased toward achieving the goal of situating lived experiences and narratives in the science curriculum. Therefore, questions were eliminated or added with this goal in mind, and to minimize the strain of additional questionnaires on the coursework. This study sought to understand both what PSTs gained from the treatment, which the author titled “Black Representation in the Science Curriculum (BRISC),” and the connections to the prevailing nature of science tenets in the field of science education.

Additional pre- and post-questions sought to shed light on Black representations and focused on the social and cultural embeddedness of science. While the additional questions were not specific to the content explored, the study sought to determine whether student engagement might lead to other implicit or explicit understandings about the adaptations of African Americans scientifically, socially, or culturally. Questions from the pre- and post-questionnaire on Black representation are shown in Table 2.

**Table 2:** Pre and Post Questions on Black representations.

Pre and post questions on Black representation
How can the lived experiences and narratives of African Americans be included in science? In K-12 science? In K-12 science curriculum? (Be as specific as possible. Provide examples where you can). What science concepts support the inclusion of the lived experiences and narratives of Blacks in the United States? What adaptations have African and early African Americans made when they were first taken out of Africa? What adaptations do you think early African Americans needed to make when they were first brought to the United States? How have early African Americans adapted to their environment?
Additional post questions on Black representation
What science content or concepts related to Black representation did you learn or explore? Please describe as much as you can. Include any vocabulary terms you were introduced to or explored that support science and Black representation. What science pedagogy or approaches were you introduced to or you learned that supports including Black representation in the science classroom? Explain how and why.

The additional post-instruction questions were designed to further probe PSTs’ understandings related to Black representations and science concepts and content. The questions on adaptations of Africans and early African Americans explored PSTs’ prior knowledge and possible inferences from their engagement with the content. The goal of this instruction was not to teach about adaptations as a specific content area. However, the assumption in this study was that by addressing ideas around food, and through explicit or implicit comparisons with foods in Africa and the United States, PSTs might make inferences about food adaptations from the data or might have explicit discussions about food adaptations of African Americans. This inferred adaptation could be derived from perceived changes our Black ancestors made or from being situated at an HBCU. It was considered possible that some PSTs might notice this change and others might not, depending on the question they explored in their inquiry.

### Data analysis

Data analysis used both schema theory and the FRA to characterize students’ responses. The nature of PSTs’ schema changes was defined by Marshall’s (1995) four knowledge types: identification knowledge, elaboration knowledge, planning knowledge, and execution knowledge [see table in Quinlan (2019b) for guiding questions]. Identification knowledge (IDK) attended to pattern recognition and activated schemas. If students’ pattern recognition was best characterized by keyword or general searches, this was designated as “+”; pattern recognition pointing to a specific science topic or concept, or an FRA connection, was designated as “++.” A lack of pattern recognition could be indicated by either “0,” no response, or align with what is typically characterized in VNOS-C studies as naïve or uninformed”–” to show similarities and points of departure from VNOS-C analyses. Elaboration knowledge (ELK) involved abstractions from experiences and hypotheses (E). This was also designated by “E” if elaborations could be characterized by FRA categories, or “e” if this was a general or uninformed example. An “e-” indicated an incorrect response or one that deviated from the question asked. Planning knowledge (PLK), or procedural knowledge, indicates a prior or developing schema and is designated by “P.” Lastly execution knowledge (EXK) is knowledge required to carry out final steps. A key is provided for any additional designations captured during analyses. The narratives presented in the findings were guided by the FRA to highlight relatedness to science when examining the narratives and lived experiences of Black heritage. The FRA categories were also used as analytic codes (Saldaña, 2021) to provide comparisons between pre- and post-instruction ideas using NVivo software. Some elements of content-analytic summary tables or partially ordered meta-matrices (Miles et al., 2020) were used to facilitate analyses and write memos.

## Findings

In this study, characterizing and identifying notable changes in preservice teachers’ VNOS and their characterization of Black narratives and the lived experiences of African Americans presented several challenges. First, this was their second science methods course with the instructor and researcher, focusing on both inquiry methods and using exemplars of Black heritage and lived experiences. Selected VNOS questions served as a vehicle in this vetting process because the narratives have implications for content that can be used in NOS studies. The findings, therefore, focus on nuanced changes or similarities, especially those highlighting the significance of Black heritage and lived experiences and the social and cultural nature of science, rather than solely on VNOS-C changes.

### Part 1: PSTs’ views of the nature of science (VNOS-C)

Overall, most preservice teachers (PSTs) began with appropriate views of what science is. Generally, PSTs maintained the same pattern recognition or identification knowledge (IDK) for what science is. Some PSTs provided more elaborations before, and others provided more elaborations after. The pre-responses inferred PSTs’ prior experiences in their prior spring semester Earth Science Methods and Practicum course, where each conducted a long-term inquiry into phenomena related to climate change and global warming. After their research, they performed their own related experiments. Table 3 summarizes PSTs’ pre- and post-changes in pattern recognition for the VNOS-C questions. A few illustrations follow.

**Table 3:** PSTs’ pre- and post-pattern recognition and elaborations for VNOS-C.

PSTs	What is science?		Do theories change?		Theory vs. law		Same data/ different conclusions		Use of creative imagination		Social and cultural NOS	
	Pre	Post	Pre	Post	Pre	Post	Pre	Post	Pre	Post	Pre	Post
Jema	+E	+	+E	+E	+0	++	−	−	++E	++	++E	++
Ashley	+e-	+E	+	++e-	−	−	+	++	++	++E	++e	++E
Mary	++Ee-	++E	++E	++E	+e	++Ee-	-e-	-e-	++E	++E	++e-	++E
DMP	++e	++E	++E	++E	-E	+Ee	−	−	++	++	++E	++E
Carmen	++E	++e	++	++	−	-E	−	+	++E	++	++E	+E
Jo	++e	++E	-E	− + E	++	++	+e	+e-	++e	++E	+Ee-	++E
Jordan	-e	+E	+e	+e-	+ −	−	-e	+E	+e-	++E	+e	+e
Michelle	+ee-	+e	+eA	+	+ − E	+ − E	+	0	++E	++ee-	+	++e
Nelly	+e	+e	0	+e	- E	++E	+e	-e-	+e	++E	+e	+e

−, Uninformed/incorrect ex. scientists do not use their imagination; +, Pattern recognition in keeping with a keyword search/general answer; ++, Pattern recognition (informed identification knowledge or specific example); 0-, No response; NC, No noticeable change; E, Elaborates (using examples that point to the categories of the FRA); e, Elaborates with general example or uninformed example; e-, Elaborates with incorrect example; A, African heritage/African American connection/Connections to Black people generally.

Almost all PSTs held similar or the same IDK for the tentative nature of theories before and after implementation. Generally, PSTs’ IDK indicated a more tentative place for theories than laws, as supported by the research, with three out of nine displaying more informed IDKs, four holding less informed IDKs, one incorrect, and another providing no answer. One student with a general IDK believed that scientific theories changed and provided a general elaboration knowledge (ELK), then a connection with the Black experience:

I do believe that scientific theories change. I think that the human mind is ever-evolving and changing, also our society is constantly changing. I definitely think that newer scientists can build off past theories but not every theory is set in stone. I think that it is human nature to always want to find out a why to anything in life so that is why we bother to learn scientific theories. A prime example I think is the theories during slavery that Black people were not the same as whites and that our skin is not thicker, skeletal structure is different, brain capacities are smaller, and that we were inferior beings. Obviously, that is a very dated theory that has been proven false and should not be used today.

Her post provided an uninformed general statement: “I think that scientific theories can change because the world is constantly changing.” Similarly, the findings on PSTs’ distinction between theories and laws supported the research on PSTs’ conceptions and misunderstandings about theories and laws in science.

For data conclusions, preservice teachers generally held the same or similar pattern recognition in both pre- and post- with only two PSTs displaying changes in pattern recognition. When compared with the other questions, PSTs provided the least number of elaborations about why scientists create different hypotheses about the extinction of dinosaurs. This makes sense when we consider that the PSTs did not engage with data as lengthily in this study as they did in the semester prior. The student who elaborated the most during post stated:

They are both possible because the data can be used to support both cases. It is like how different illnesses can have similar symptoms but they affect people differently (pre, Jordan).

I have not looked into this phenomena, and my teachers never really went deeply into it. I would say these different conclusions are possible because both sides of the argument have evidence to back up their claims. May it be physical evidence, written evidence, or similar experiences that back up their claim they have something to make both claims credible (post, Jordan).

It is possible that she abstracted from the exercises on argumentation that included looking at claims and evidence. Her response also reflected her meta-thinking about whether or not she was taught it or looked into it.

#### Scientists’ use of imagination

Preservice teachers’ responses to whether scientists use their imagination showed stark contrasts with their responses to the scenario on dinosaurs. Concerning the use of imagination and creativity, their explanations abstracted from scientific practices and processes, whereas they had difficulty articulating the role of data. The results showed that almost all PSTs maintained their pattern recognition for scientists’ use of their imagination. Most PSTs explicitly pointed to the planning and designing of an experiment as a place where scientists use their imagination (7 out of 9), and they maintained this IDK either explicitly or implicitly in the post-condition. Some mentioned the importance of imagination in hypothesis generation, sometimes seeming to add it as an addendum to planning where their pattern recognition might infer their prior experiences doing an implementation experiment that aligned most closely with their research paper interest on climate change—a high cognitive demand task which required a great deal of scaffolding and creativity. In some cases, their ELK was more detailed in the pre-VNOS-C even as their IDK or pattern recognition remained the same, such as Michelle’s pre-(++E): “I think that scientists use creativity and imagination when coming up with a topic or experiment, how they will execute it, and what they gather from the results. Also during the hypothesis stage,” and post- (++ee-): “I think scientists do use their creativity in planning and design. Scientists have to have a level of creativity because they are researching something that has never been done before or seen in that way.”

#### Social and cultural NOS

Eight out of nine PSTs (8 of 9) were explicit about their belief that science reflects social and cultural values in the pre-VNOS-C, and all PSTs were explicit about this belief in the post-condition. Of the eight PSTs who were explicit about this belief, five displayed an informed IDK (++) in the pre-VNOS-C, and six displayed an informed pattern recognition in the post VNOS-C. Most informed perspectives were accompanied by ELKs that reflected elements of the social-institutional dimension of the FRA, and which also implicated a role for the cognitive epistemic. DMP’s responses pointed to both the scientific ethos in the pre, and the role of social values or social ethos in the post:

I believe science i don’t social and cultural values because many scientists only focus on what they learn from professors of their same background or text from those they relate to; other scientists prefer to take ideas from many different scientists/sources to come up with new and modern findings (pre, DMP).

Science reflects social and cultural values, in my opinion, because people use their prior knowledge to make some findings. An example is someone who is trying to discover a way to create a new skin ointment. They may have used coconut oil in their life to heal their skin and incorporate that into their product (post, DMP).

Others were more explicit about the social role of the cognitive epistemic by pointing to the role of biases or prior knowledge. The student who displayed the most drastic change in belief provided ELKs to support both perspectives:

I believe that that science is universal because facts are analyzed and proven to apply beyond social and cultural boundaries. One thing that comes to mind is chemistry. Interactions between certain metals, alloys, and other elements are concentrated the same all over the world to make jewelry. If science was based on social values it would be made and used differently (Pre, Jo).

I believe that science reflects social and cultural values due to the lack of representation seen by African American scientists and engineers. If science was universal and accepting, there would be great advances in STEM due to appreciating all scientific inquiry. However, to this day, there can be seen a disconnect in published articles, books, and documents that reflect science. Social and political biases are often woven into science making it difficult for every student to learn (post, Jo).

In general, most PSTs’ pattern recognition (IDK) for the social and cultural NOS were consistent, with differences in their supporting ELKs. For example, Ashley first provided an IDK that generally pointed to practices: “Science reflects social and cultural values because some cultures have different practices when researching [IDK]” (pre, Ashley). Her post-ELK then explicitly abstracted from an example she learned in class: “I believe that science can reflect social and cultural values. [IDK] For example, when learning about the production of cane syrup (which is a product widely used in southern culture) I was also able to learn about the negative effects it has on the health of those in my community. [ELK]” (Ashley, post).

### Part 2: Black representation in the science curriculum

Overall, the results showed that the questions on Black representation in the science curricula provided more insights into the social-institutional system dimensions than into the cognitive–epistemic system dimensions. Post-questions #11 and 12 were added to further probe what science content, concepts, or science pedagogy PSTs learned. These additional post-hoc questions were helpful in elucidating PSTs’ potential schemas for including the lived experiences and narratives of Black heritage. Table 4 displays a comparison between the pre- and post-scores.

**Table 4:** PSTs’ pre- and post-changes to Black representations in the science curriculum questions.

PSTs	Including experiences, narratives of African Americans		Science concepts for lived experiences & narratives of Blacks in U. S.		Adaptations of Africans and early African Americans Question #9/ #10		Science content/ concepts/pedagogy learned on Black representation. Question #11/#12
	Pre	Post	Pre	Post	Pre	Post	Post only
Jema	-e-	+	+	+	0 / -	+/ ++	0/0
Ashley	+	+E	0	+	+ / +	+/ +	++ / ++
Mary	+E	+E	0	++E	+ / ++	+/ ++	+P / +P
DMP	++E	++EE	++E	++	+e / +	+ / +	++ / +P
Carmen	+E	++E	0	+ e	+ / +	+ / +	++ / +P
Jo	+E	+E	- e-	++E	+ / +	++E / +	+ / ++P EP
Jordan	++E or +E	++E or +E	+	++E	+ / +	++ / +	+ / +
Michelle	++E	++	++E	++E	+ / +	+ / +	++ / +
Nelly	+e	+e	-e-	−	++ / +	++ / +	+e / +e

-, Uninformed/incorrect ex. explicit about not knowing, general answer in keeping with keyword searches; +, Pattern recognition (identification knowledge that points generally to scientists or contributions). General statement that does not point to specific example; ++, Pattern recognition (informed identification knowledge or specific example) especially a specific example from the course implementation; E, Elaborates (using examples that point to the categories of the FRA); e, Elaborates with general example or uninformed example; e-, Elaborates with incorrect example; NC, No noticeable change; A, African heritage/African American connection/Connections to Black people generally; P, Procedural Knowledge; 0-, No response.

#### Lived experiences and narratives

Most PSTs (7 out of 9) held similar identification knowledge for how to include the lived experiences and narratives of African Americans. Their pattern recognition was consistent with commonly held perceptions for including representations using the contributions of Black scientists or other professionals in the field of science or discussing the contributions of African Americans to science and/or society. While all PSTs’ IDK were maintained, those with modified IDKs displayed connections with or explicitly attended to the new content learned: “I think lived experiences and narratives of African Americans can be included by looking into topics that affect the community” (pre-IDK, Carmen) to “Lived experiences and narratives of African Americans can be included in science and K-12 science by looking at the effects food sources have had on African Americans” (post-IDK, Carmen). PSTs’ elaborations abstracted from what they learned. One student’s pre-ELK (++E) pointed to what she learned in a previous science methods course where we discussed Dr. Charles Drew. (Charles Drew was an African American doctor most noted for his practical work preserving blood, which led to our current wide-scale use of blood banks). However, her post-ELK seemed to abstract from information in the current investigation. Both were designated as “++EE”:

The experiences and narratives of African Americans can be included in science by studying the land of these individuals and learning about how they affected the architecture and geography of the world. In K-12 science teachers can implement studies and ideas from black scientists, such as Dr. Charles Drew. In K-12 science curriculum students can learn about the anatomy of black bodies in comparison to other body types to understand genetics (pre, DMP).

The lived experiences and narratives of African Americans can be included in science by conversing with people who have been able to make advancements in black communities. In K-12 science teachers can teach about African health and disparities. In the curriculum there can be an implementation that caters to teaching about how African practices have influenced American science advancements. An example could be how African scientists may have used their native healing methods to discover a medicine that has helped Americans (post, DMP).

Her post described procedures or PLK that informed the creation of the current treatment, where members of the Gullah/Geechee community were interviewed by the researcher, thus drawing parallels to what could be done. Other specifics that pointed to practices and healing inferred abstraction and learning from this study treatment.

Overall, PSTs’ responses to including the lived experiences of African Americans pointed to the social values of science that pertained to addressing community needs and the importance of equity in intellectual authority by sharing the contributions of African Americans in science. The findings also highlighted the importance of how PSTs interpreted the questions, especially prior to learning about the topic. In the pre-condition, one student explicitly stated not understanding what was meant by the question on including lived experiences and narratives.

#### Science concepts for inclusion

The importance of having a prior schema for what the question was asking was especially true for the question on what science concepts and content supported the inclusion of the lived experiences and narratives of Black people in the U. S. Three PSTs left this question blank, four were coded as either uninformed or incorrect, and two displayed more informed views prior to treatment. One, coded as (+) or as a general statement, was explicit about not understanding the question even though she displayed some IDK:

I am little confused by the question but eugenics and natural selection are topics that can potentially revolve around African Americans (+) (Jordan, pre).

Concepts that involve hunting and gathering dive into the inventions people of color created such as the bow & arrow and boats. Astronomy is a science concept I am familiar with that supports the experiences of African Americans. Historically, constellations, stars, and planets were used to make calendars and systems of time keeping. In addition, they were used for navigation. As many know, Harriet Tubman used the North Star to guide her on the Underground Railroad (++EE) (Jordan, post).

The specificity she provided here for the ELK elaborated on her prior schema for the social-institutional system dimensions of science, namely the injustices in science and connections with history. This was especially true for the two PSTs who displayed drastic changes in perspectives. One abstracted explicitly from the treatment, and another’s abstraction could be inferred. For example, Mary, who had no response during the pre-condition, indicated explicitly in the post:

An example of this occurring was during our lesson on the African American Gullah Geechee. Their scientific and medicinal ways of living are not typically thought of as “scientific” (even though they are). Including the experiences of the Gullah Geechee helps African Americans students see the many different ways science is seen in various African American communities (post, Mary).

The student whose pre-condition was both incorrect and elaborative, focused on community impact during the post as shown below:

STEM has been emphasized more within K-12 schools and curriculum in underdeveloped urban schools around the United States. This inclusion of STEM is building the interests of young black youth to pursue STEM careers in the future (Pre, Jo).

I would say that the science of food safety reflects the lived experiences African Americans go through in the US. As we spoke about food security and accessibility in the class throughout the year, economic difficulties lay heavily within low-income and underserved communities. This community population is mostly made of African Americans and other non-European races who are discriminated against (Post, Jo).

Her post-pattern recognition for science concepts and science content was explicit about what information was abstracted from the discussions in class, as she made connections with political power structures and financial system categories of the FRA. Table 5 lists the inquiry questions on NRC’s *Lost Crops* that PSTs briefly explored in the course.

**Table 5:** PSTs’ inquiry questions.

PSTs	Inquiry questions for research
Jema	Should women take moringa during pregnancy to aid in child development?
Ashley	Are fruits such as melon and watermelon an alternate supply for water/hydration? What other nutrients do they supply?
Mary	Why were sugarplums able to sustain people despite it being a low-calorie fruit?
DMP	What other foods can provide the nutrients found in these lost grains?
Carmen	If sorghum and corn come from the same plant family how do they affect people’s diets differently?
Jo	How does the nutritional composition of shea and cocoa butter compare in terms of benefits for the skin?
Jordan	Moringa is a very nutritious plant. Due to its beneficial qualities, is it used for anything else in addition to consumption? In addition, what methods does the plant undergo before it is eaten or potentially used?
Michelle	How do these fruits compare to today’s fruits.
Nelly	Why are nutrient dense African fruits not popular to consume in American culture?

#### Adaptations of African Americans

The first question on adaptations did not elicit responses specific to science but had implications for the FRA. Preservice teachers pointed to adaptations to language, weather, or food, which could imply connections to science. However, science connections were not explicitly made. On i dont hand, the second question on adaptation, which pointed specifically to the environment, drew out some responses that were characterized as informed because more specific ELK were provided. For example, the student that showed the least amount of progress in the first question displayed more informed IDK for the second question on adaptation. In the pre-condition she noted: “I am not sure of adaptations that Africans made but I’m very interested to learn more” (pre, Q9) and “Adaptations that would help them day to day life and get their work done easier” (pre, Q10). Question 10 seemed to provide continued sense-making for question 9. Then in the post-condition she wrote, “Medicines, food, etc.” (post Q9) and “Medicine, they learned about the herbs around them” (Post Q10). While the pattern recognition or IDK were the same in both questions, question 9 provided keywords whereas question 10 made connections with what was discussed.

Overall, PSTs displayed the same or similar IDK in their responses throughout. One ELK showed the probing effect of the subsequent question. In the pre-condition she noted: “When they were first taken out of Africa, Africans and early African Americans adapted by language to communicate in different areas of their life including science and math” (Q9). Her response to question 10 seemed to elaborate on this while providing similar IDK: “They needed to make adaptations to the way they converse, think, learn, and were trained. Being forced to be slaves their environment was something that they had to work around to create self-identity.” Thus, question 10 provided confirmation for PSTs’ pattern recognition as well as supported a probing effect. For example, this same student provided more elaboration for the first question and less ELK for the second, which could infer that she had expressed all she could or felt she needed to:

Africans used creativity and engineering as a means of adaptation in order to survive. Many inventions made then have translated into modern innovations, no matter how big or small. As slaves, Africans and early African Americans had to use what they had in order to make their lives easier. In doing so, they began to learn more about science in their own investigatory ways (Post, Jo, Q9).

Thus, the post response to Q10 seemed to provide a summation for Q9.

“They needed to adapt to using materials to gather food, water, and shelter” (Post, Jo, Q10).

Her explicit connection to STEM in the post-condition should be noted. Moreover, PSTs’ responses to questions on adaptations pointed to the social utility and social value of science for the lived experiences of people of Black heritage.

#### Science content and pedagogy

The two questions, #11 and #12, on science content and pedagogy were added only in the post-condition to probe for additional insights into implicit abstractions about science pedagogy and concepts. The findings pointed to two major outcomes. First, they inferred the complexity of the questions and the difficulty of drawing out implicit information. Second, the findings showed that PSTs either attended to procedural knowledge, designated as “P,” or to non-procedural information. Thus, P, or PSTs’ IDK or pattern recognition for science pedagogy, inferred possible schema acquisition or planning knowledge (PLK). For example, for both questions, one student responded by stating what she learned: “I learned about the effect sugar cane has on putting the Black community at risk for diabetes” (Ashley, Q11) and “I learned about how fruits, vegetables, and grains provided an alternative source of nutrients for those in the black community” (Ashley, Q12). In both cases, her pattern recognition was designated as ++ because of her specific and informed knowledge, even though the latter did not directly point to science pedagogy. Preservice teachers whose responses to the question on science pedagogy indicated procedural knowledge (P) identified “making meaningful connections” (Mary) and “incorporat[ing] science by studying black cultures” (Carmen). Only one student provided an elaborative “P” that could indicate PLK: “I was introduced to connecting African/African American history into the classroom that emphasized Black engineering. In addition, engineering was also a key concept that created a space where PSTs could use their higher order thinking skills to begin an inquiry process” (Jo). She ended her response by alluding to the inquiry she conducted.

While PSTs’ responses attended to the social utility of science for the Black community and to the general social–epistemic dimension, the inclusion of science concepts, content, and pedagogy could also point to the cognitive–epistemic system dimension. This is because it changes the goals, values, and objectives of the inquiry or inquiry process, while providing differences in “viability and explanatory power,” novelty, and other characteristics of the cognitive–epistemic system. For example, scientific practices might include using “historical data” to create specific arguments and reasoning, and to generate different outcomes. Identifying the inquiry process used also highlighted the importance of methodology (Quinlan, 2021).

## Discussion

### Rationale and motivation for this study

The goal of this research was not to vet the VNOS-C, as this has already been done. Therefore, selected VNOS-C questions were used rather than the entire instrument. This study also assumed that an environment that already utilizes the lived experiences and narratives of Black heritage, such as might be found at an Historically Black College and University (HBCU) rather than at an Historically White College and University (HWCU), would influence the nature of science, especially as it relates to its social and cultural embeddedness. The significance and groundbreaking nature of this work lies in providing both content and processes that capture the three criteria Ladson-Billings uses to define culturally relevant pedagogy: academic success, cultural competence, and critical consciousness. In this research, students had the opportunity to explore questions about the cultural scientific practices of their Black heritage. Ladson-Billings (1995) notes that “students must develop and/or maintain cultural competence” (p. 160). The need for cultural competence or cultural relevance is not discussed in relation to science subject content knowledge or the nature of science. Moreover, how understandings about the sociocultural nature of science are organized in memory or students’ prior schemas might reveal a different orientation that lends significance and nuances to students’ social and cultural VNOS-C changes. Science or NOS studies may change the approach or processes but not the content. Ladson-Billings (1995) notes that to achieve cultural relevance, “students must develop a critical consciousness through which they challenge the status quo of the current social order” (p. 160). This study addresses the need to challenge how Black heritage knowledge and cultural knowledge are positioned in science content while engaging students in practices.

This study was driven by the motivated thinking and goal to situate in the literature and show the usefulness of using the science capital, narratives, and lived experiences of Black people and of Black heritage as a context for inquiry explorations that facilitate understandings of science and of the nature of science. Molden and Higgins (2005) provide evidence which shows the impact of motivated thinking or what they describe as the influence of the heart on the mind. They found that people are motivated by thinking which promotes positive attributions about themselves and that this influences how they evaluate evidence and what evidence they look at, the strategies they use in research, and the extent to which their goals are oriented toward closure or accuracy. Ladson-Billings et al. (2024) discuss their observations that attempts to bridge the gap, whether couched as culturally relevant or as “critical language awareness” (p. 474) resulted in “reproducing the kinds of nefarious white supremacist ideologies that sometimes even well-meaning teachers and researchers held” (p. 474). Thus, in keeping with the gate-breaking nature of this research, this work was never couched to appeal either to Eurocentric goals nor to theorizing which lacked the critical thinking needed to meaningfully situate Black narratives and lived experiences in meaningful ways, using best practices. Rather, this work creates multimedia content and other exemplars which capture the lived experiences and narratives of Black people, and which could be used for classroom engagement (see Quinlan, 2023).

By using schema theory and the FRA rather than only the standard characterizations of VNOS-C responses as naïve or informed, this study provides insights into nuances in students’ changes. The significance of this approach was to preserve what LaPoint et al. (2006) describe as the “integrity-based ethos” which characterizes their Talent Quest Model work with Black children of low-income. According to LaPoint et al. (2006) this “integrity refers to the complexity, coherence and meaning contained in students’ life experiences. These experiences are, thus, not simply characterized by deficits, inadequacies, pathologies consistently maligned in academic publications as well as multimedia reports without balanced and asset-based assessments or characterizations” (p. 375). This study instead points to the significance of new meanings, perspectives, and new ideas to attach to the NOS, and which the preservice students connect with the content they explored, influenced by their lived experiences.

### Part 1: views of the nature of science (VNOS-C)

Overall, while the findings showed that PSTs held similar or the same pattern recognition in pre and post, increased modifications were observed in PSTs’ ELK and in how they abstracted from their experiences. Schema theory showed the information PSTs connected with and attended to, thus illustrating the fluid nature of PSTs’ ideas and experiences. Preservice teachers come with prior schemas about the social and cultural impacts of science. While a significant change in this belief is not reflected in the results, the use of IDK and ELK reflects nuances that capture changes. Generally, PSTs’ uninformed IDK are characterized by keyword searches or what Offredy and Meerabeau (2005) describe as “forceful features.” This is supported by research which showed that PSTs search their memories for associations with ideas, and their lack of elaborations often indicates a lack of schemas or transfer for the ideas (Quinlan, 2019b, 2020). This makes sense when we consider that the PSTs were elementary education majors and non-science majors. Furthermore, it could be inferred that PSTs’ prior long-term project during the previous spring semester led to changed and more permanent schemas which they abstracted from, compared with this short inquiry exploration. This in no way indicates a lack of viability of the context using Black science capital, heritage, and lived experiences. This is supported by research which shows that not only does context matter but also the science approach and the use of explicit reflective strategies (Hanuscin et al., 2006). Thus, next steps should focus on making explicit connections to NOS and determining what ideas reflect how the lived experiences and narratives of Black heritage and science capital intersect with the nature of science.

Construction of treatment should consider not only how theories and laws intersect with Black lived experiences but also where these intersect with science. Preservice teachers generally hold a schema for theories and laws that bear important connections to both Black lived experiences and to science. Therefore, this is an area with great potential for expansion that could inform the social-institutional dimensions of science. Theorizing related to Black lived experiences often points to injustices even as they are connected to science.

Preservice teachers’ concepts about the use of data are supported by their limited engagement time with data. At the same time, they used creativity to come up with questions, to determine where the data came from, and to place them into tables, which were reflected in both their pre- and post-agreement that scientists use creativity. Engagement time with the data is important and necessary for any change to occur in perspectives about data.

### Part 2: Black representation in the science curriculum

The results show that the PSTs progressed toward more informed views about including the lived experiences and narratives of Black heritage, views that were informed by the treatment itself. The PSTs had a general prior schema that pointed to including Black scientists, which is a commonly held conception. Overall, PSTs displayed more informed views about including the lived experiences and narratives of Black heritage after implementation.

The most significant change occurred in PSTs’ perceptions about science concepts that support the lived experiences and narratives of Black heritage. This is supported by the inquiry exploration treatment where each PST explored a science-related topic, with some more closely related to science than others. Overall, the questions about science concepts abstracted from experiences were not reflected in the VNOS-C.

While the nature of science does not address adaptation as either a social or cultural NOS, the concept of adaptation is important to the lived experiences and narratives of Black heritage. While adaptation in science could broadly point to biological or behavioral adaptations, research points to the psychological and hence behavioral adaptations when scientists reorient themselves in response to microaggressions they experience (Brown et al., 2016). Judith Carney’s work extensively documents the nature of adaptations that early African Americans made, such as the nature of technology transfer for rice cultivation from West Africa to South Carolina and Georgia (Carney, 1996), along with food adaptations, bringing their assets with them from Africa to the United States (Carney, 2001). However, the question on adaptation mainly elicited non-scientific but science-related responses about the lived experiences of Black heritage. This points to the need to attend to these adaptations explicitly in the content knowledge, as well as the need to add content which helps students understand the various adaptations of their ancestors. Including ideas about science in the question elicited more responses related to science. Thus, the role of questions and questioning is also implicated in this research.

Planning knowledge (PLK) and potential schemas are more important when including science pedagogy. It is a safe assumption that attending to science pedagogy calls for PLK and provides a better indication for procedural knowledge retained or potential transfer from PSTs’ experiencing the context and inquiry to their implementations in future classrooms. This is supported by Leden et al. (2015) findings that it was easier for teachers to articulate what the NOS is than it was to talk about teaching NOS tenets. Transfer of learning to other contexts requires repetition for schema acquisition or planning knowledge (Quinlan, 2019b, 2020).

## Limitations

Science education research needs to pay closer attention to including the lived experiences and narratives of Black heritage as a context and a way of knowing to understand the NOS. The lived experiences of Black people are defined by the sociocultural embeddedness of science and could provide an explicit approach to understandings about NOS. However, an explicit approach that is inclusive of Black heritage should pay more attention to science pedagogy that not only reflects an explicit approach but also the scientific practices and long-term inquiry with scientists that are found in NOS studies. This research implementation focused on a brief inquiry exploration framed using the 5E model rather than the tenets of NOS. For example, attention to ideas about theories and laws using Black heritage as a context alongside the cognitive–epistemic dimensions might create a more comparative implementation approach.

More changes might have been observed if PSTs had no prior experiences conducting inquiry in their science methods course or if they had not engaged in a long-term inquiry exploration the semester prior to this study. As an exploratory study, no explicit or sustained connections were made to science pedagogy or content, even though this question was asked and even though PSTs conducted inquiry explorations. Furthermore, no interviews were conducted to probe for or clarify perspectives from the questions. This would be an important next step to create a parallel to NOS studies that use other contexts and to clarify PSTs’ responses to both the VNOS-C and the BRISC questions. However, it is important to note that this type of probing comes with additional assumptions about PSTs’ meta-thinking abilities. Nevertheless, the process is helpful in elucidating and for reliability. Instead, the PSTs’ experiences were inferred and triangulated by looking at their inquiry explorations.

### Pros and cons of using multiple frameworks

The larger research study did not begin with the multiple frameworks used in this research paper. Rather, their use emerged and was chosen because the frameworks provided additional perspectives and vetting for a new context in the science education literature already saturated with NOS studies. While the FRA was used to explain the data, one consequence of using the FRA to bring attention to and make connections with NOS is that the framing appears superficial. If the FRA had focused on describing implementation and treatment, then richer discussions about scientific practices would have been provided, but this would change the focus of the findings given space limitations. Instead, the findings would have focused on the resemblances between science and an exploration using Black heritage (Quinlan, 2024a) rather than on NOS outcomes. Rather, the FRA drew attention to where the social dimensions of NOS were apparent or inferred. Schema theory enabled a focus on epistemology as it related to context, prior knowledge, and NOS findings. The FRA was important in making a case for not ignoring social and cultural contexts within content to be considered alongside cognitive dimensions. Schema theory overlapped with the FRA in considering both the cognitive and the social and cultural impacts on knowledge structures, even though this research focused on the NOS outcomes and less on the context and content for implementation.

## Conclusion

This study situated the lived experiences and narratives of Black heritage and of African Americans in the nature of science research literature. This was especially important because nature of science research mainly captures Western contexts or White European histories and lived experiences. Furthermore, NOS treatment mainly reflects the experiences of historically White PSTs, or Black PSTs are not reflected in the literature (Walls, 2016). Situating this work in conversations about the nature in science is in of itself gate-breaking and accomplishes the goals of culturally relevant pedagogy and Paris (2012) Culturally Sustaining Pedagogy by “creating a pedagogy that explicitly embodied resistance to the status quo and encouraged marginalized communities to fight for their linguistic and cultural sovereignty” (Ladson-Billings, 2021, p. 351). Nature of science research is pivotal to discussions about scientific practices and the role of the NGSS, and the importance of this work is echoed in the 2024 National Association for Research in Science Teaching conference theme, “Science Education For The Rest of Us.”

It is also important to note that this work could not be accomplished by doing what Ladson-Billings (2021) observed as an unfortunate occurrence: assigning teachers articles about culturally relevant pedagogy without demonstrating what this looks like. This work was accomplished through, and continues to consider, teacher predispositions, opportunities for self-reflection, inquiry explorations into Black heritage and lived experiences, best practices in science education pedagogy, and new experiences for teachers and students alike. These elements provide critical thinking opportunities and model higher-order thinking and learning (Quinlan, 2024a). As discussed in Quinlan (2024a), this work emerges from a need to understand what these theories are and mean for the classroom setting and for Black peoples, thus distinguishing between knowing *what* and knowing *how* to get there, not just for the researcher but for students at any stage of education.

This work echoes the call from researchers of culturally sustaining pedagogies, who were themselves prompted to ask different and more meaningful questions (Ladson-Billings et al., 2024). Ladson-Billings et al. (2024) discuss the need to go beyond asking “what’s *wrong* with Black children” (p. 472) or “‘How can I fix it?’” (p. 473) to asking “What was right. *what does that look like?* (p. 472*)* and “how can we join, revitalize, return, deepen and grow?” (p. 473). Similarly, the larger and groundbreaking goal of this work asks: *How can Black heritage and lived experiences be meaningfully included in STEM curricula, content, and pedagogy in identity-forming and affirming ways, to create competitive and cutting-edge learning experiences for Black children?* Let this be the beginning of conversations in science education research to define what contexts and content capture the science capital and lived experiences of Black and Indigenous heritage, and from these same perspectives, using vetted and best practices in science education pedagogy. Moreover, nature of science studies should provide space and opportunity for Black, Indigenous, and other underrepresented groups to contribute additional voices and ways of knowing that provide accurate content and scholarship, representation, and inclusion—all of which King and Swartz (2016) note are culturally informed principles for content and pedagogy.

## Data Availability

The datasets presented in this article are not readily available because this includes student data that I will continue to use in a follow up to this project which focuses on curricula. Some of the treatment data will be made available there. Further work is also needed to anonymize. Requests to access the datasets should be directed to Catherine Quinlan; cquinlan@nccu.edu; see website for updated contact, https://orcid.org/0000-0002-9493-3164.
